# Tris(2-hydroxyethyl)ammonium-Based Protic “Ionic Liquids”: Synthesis and Characterization

**DOI:** 10.1021/acs.jced.4c00024

**Published:** 2024-04-24

**Authors:** Emilia Tojo, Alexandra Cáceres, Alba Somoza, Carlos A. Pena, Ana Soto

**Affiliations:** † Department of Organic Chemistry, Faculty of Chemistry, Universidade de Vigo, 36210 Vigo, Spain; ‡ CRETUS, Department of Chemical Engineering, Universidade de Santiago de Compostela, E-15782 Santiago de Compostela, Spain

## Abstract

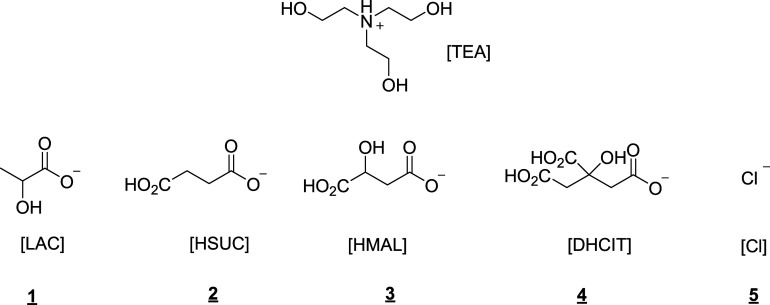

The proton transfer associated with the synthesis of protic ionic liquids (PILs) is often incomplete, meaning that the parent compounds may coexist with the ionic species. However, PILs are proposed for many applications as pure compounds without analysis of their ionicity. This work focuses on tris(2-hydroxyethyl)ammonium-based PILs with lactate, hydrogen succinate, hydrogen malate, and dihydrogen citrate anions. The interest of these anions lies in their low toxicity and capacity to disrupt the hydrogen-bonding network inherent to biopolymers. To improve current synthesis methods of this kind of PILs, which frequently lead to impurities derived from decomposition of reactants, working in the absence of solvents and at moderate temperatures is proposed. Through NMR studies, the ionicity of these systems was found to be low, from 20% to 86%, so the widely used term “ionic liquid” is not rigorous and must be used with caution. The un-ionized acid and base species coexist with the corresponding ionic forms, and this has to be considered in the studies involving these chemicals. The thermal characterization of the PILs was carried out. The influence of the anion on the thermal stability was found to be low. Isothermal thermogravimetric analysis showed that mass loss of these PILs starts at temperatures close to 350 K.

## 1 Introduction

Protic ionic liquids (PILs) are formed by proton transfer from an acid to a base. In an ideal scenario the transition is complete, and the system is only composed of ionic species. However, it is known that the proton transfer is often not complete. Consequently, both the acid and base species may coexist with the ions. Furthermore, there is the possibility of aggregation and association, leading to the formation of complexes involving either ions or neutral species.^1^ MacFarlane and Seddon^2^ looked over the term “ionic liquid” and suggested only consider a pure ionic liquid when less than 1% of the neutral species are present. There are several methods to determine the ionicity of protic ionic liquids such as NMR, spectroscopy, the use of p*K*_a_ values of the precursors in water, changes in thermal properties or ionic conductivity, etc.^1^ However, the term PIL is frequently used in the literature without an analysis of ionicity. Furthermore, high purity is associated with these products and they are usually considered as just one compound, for instance, in equilibrium studies.

2-Hydroxyethylammonium (or ethanolammonium)-based PILs have been proposed for several applications. Yuan et al.^3^ tested several hydroxyl ammonium ILs combined with formate, acetate, and lactate anions for SO_2_ absorption. They found that solubilities at ambient pressure were quite high, and highlighted the possibility of recycling and using the PILs repeatedly as an advantage over traditional solvents. Moreover, SO_2_ could be recovered and used as a source of sulfur. Triethanolamine acetate, diethanolamine chloride, and their corresponding Pd(II) complexes showed low antibacterial but good antifungal activities.^4^ Kondratenko et al.^5^ called attention to the biological activity of triethanolammonium salicylate due to its unique atrane structure characterized by the presence of three trifurcated hydrogen bonds.

The use of ILs in the treatment of lignocellulosic biomass, within the biorefinery context, is on the cutting edge of scientific research due to the efficiency of these salts in biomass dissolution, selectivity in component separation, recyclability, absence of atmospheric contamination, safety advantages, and alignment with green chemistry principles. So it is not surprising that ethanolammonium PILs have also been proposed for this application. The combination of 2-hydroxyethylammonium, bis(2-hydroxyethylammonium) and tris(2-hydroxyethylammonium) cations with malate, malonate, succinate, glycolate, and lactate anions resulted in excellent solvents (as pure compounds or mixed with water) to dissolve Kraft lignin at low temperature. It was also shown that the anion plays a key role in the process, the effect of the cation being secondary.^6^ In another study,^7^ the same authors worked with carboxylate (formate, acetate, propionate, hexanoate, octanoate) anions and showed the nonderivatizing character of the PILs in the application.

2-Hydroxymethylammonium, 2-hydroxyethylammonium, 2-hydroxydimethylammonium, and 2-ethylhexanoate PILs were proposed as lubricants or lubricant additives in steel–steel and steel–aluminum contacts.^8,9^ Even when the percentage of ionised species in these compounds was not determined, the ionic nature (determined through conductivity measurements at room temperature) was related to the lubricant character. The highest ionicity corresponded to the poorest friction and wear behaviors due to tribocorrosion reactions on the steel surface. 2-Hydroxyethylammonium oleate also proved to be a good lubricant for aluminum-forming processes.^10^

**Figure 1:**
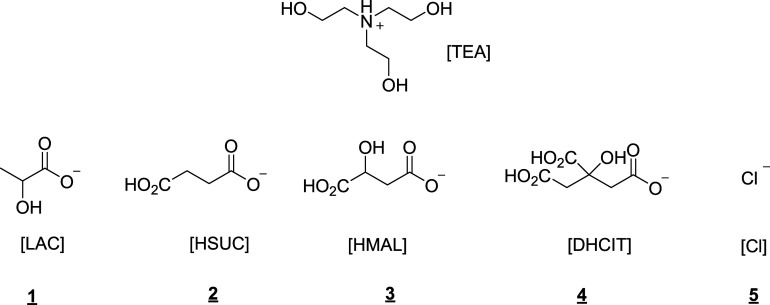
Structure of the salts synthesized. **1**: [TEA][LAC]; **2**: [TEA][HSUC]; **3**: [TEA][HMAL]; [TEA][DHCIT]; and **5**: [TEA]Cl.

This work focuses on tris(2-hydroxyethyl)ammonium ([TEA])-based PILs with lactate ([LAC]), hydrogen succinate ([HSUC]), hydrogen malate ([HMAL]), and dihydrogen citrate ([DHCIT]) anions (Figure 1). Tris(2-hydroxyethyl)ammonium was selected as cation due to its potential biodegradability, and the anions due to their capacity to disrupt the hydrogen-bonding network inherent to biopolymers. A critical analysis of possible reaction conditions in their synthesis is carried out. ^1^H MNR spectroscopy is used to determine the percentage of ionised species, and differential scanning calorimetry (DSC) and thermal gravimetric analysis (TGA) are used to investigate the thermal behavior of the synthesized compounds.

## 2 Experimental Section

### 2.1 Chemicals

#### 2.1.1 Reagents

Reagents used for the synthesis of the selected salts were supplied by Sigma-Aldrich for tris(2-hydroxyethyl)ammonium (also called triethanolamine) (≥99%), dl-lactic acid (USP testing specifications, 99%), succinic acid (≥99%), dl-malic acid (99%) and citric acid (99%), by PanReac for methanol (≥99.9%), and by Acros Organics for hydrochloric acid (37% solution in water). These chemicals were obtained from the commercial supplier and employed without further purification.

#### 2.1.2 TEA Salts

The synthesis of the PILs was carried out according to an optimized procedure as explained below (section 3.1). When the acid was a liquid (lactic acid), it was added dropwise to an equal molar amount of tris(2-hydroxyethyl)amine cooled in an ice-bath under argon. When the acid was a solid (succinic, malic and citric acid), an equal molar amount of tris(2-hydroxyethyl)amine was added dropwise to the acid cooled in an ice-bath under argon. The mixture was allowed to stir at 323 K until completion of the reaction, monitored by thin layer chromatography (t.l.c.) using silica gel 60 GF-254 aluminum sheets and CHCl_3_-MeOH 9:1 as eluent. Their structures were confirmed by ^1^H and ^13^C NMR spectroscopy (spectra are provided in Supporting Information, SI). From the NMR spectra, a purity ≥99%wt was estimated for all the synthesized salts. To avoid the presence of water, the reaction products were freeze-dried by lyophilization.

##### Tris(2-hydroxyethyl)ammonium Lactate [TEA][LAC]

Reagents: tris(2-hydroxyethyl)ammine (1.08 g, 7.19 mmol) and lactic acid (0.54 mL, 7.19 mmol). Reaction time: 15 min. The product was obtained as colorless crystals.^11^ Yield ≥ 99%. ^1^H NMR (400 MHz, DMSO-*d*_6_, ppm, δ): 5.42 [bs, O*H*], 3.95 [q, 1H, *J* = 6.8 Hz, C*H*CH_3_], 3.48 [t, 6H, *J* = 6.0 Hz, C*H*_2_OH], 2.70 [t, 6H, *J* = 6.0 Hz, NC*H*_2_], 1.20 [d, 3H, *J* = 6.9 Hz, CHC*H*_3_]. ^13^C NMR (100.6 MHz, DMSO-*d*_6_, ppm, δ): 177.49, 66.56, 58.86, 57.23, 21.15.

##### Tris(2-hydroxyethyl)ammonium Hydrogen Succinate [TEA][HSUC]

Reagents: tris(2-hydroxyethyl)ammine (20.94 g, 138.9 mmol) and succinic acid (16.57 g, 138.9 mmol). Reaction time: 15 min. The product was obtained as a white solid.^12^ Yield ≥99%. ^1^H NMR (400 MHz, DMSO-*d*_6_, ppm, δ): 3.47 [t, 6H, *J* = 6.0 Hz, C*H*_2_OH], 2.68 [t, 6H, *J* = 5.9 Hz, NC*H*_2_], 2.38 [s, 4H, C*H*_2_C = O]. ^13^C NMR (100.6 MHz, DMSO-*d*_6_, ppm, δ): 174.54, 58.71, 57.13, 30.03.

##### Tris(2-hydroxyethyl)ammonium Hydrogen Malate [TEA][HMAL]

Reagents: tris(2-hydroxyethyl)ammine (17.86 g, 118.48 mmol) and malic acid (16.05 g, 118.48 mmol). Reaction time: 1 h. The product was obtained as colorless crystals. Yield ≥99%. ^1^H NMR (400 MHz, DMSO-*d*_6_, ppm, δ): 6.33 [bs, O*H*], 4.01 [dd, 1H, *J*_1_= 7.8 Hz, *J*_2_= 5.6 Hz, C*H*OH], 3.63 [t, 6H, *J* = 5.6 Hz, C*H*_2_OH], 3.02 [t, 6H, *J* = 5.7 Hz, NC*H*_2_], 2.55 [dd, 1H, *J*_1_= 15.5 Hz, *J*_2_= 7.8 Hz, C*H*_α_C = O], 2.34 [dd, 1H, *J*_1_= 15.6 Hz, *J*_2_= 5.6 Hz, C*H*_β_C = O]. ^13^C NMR (100.6 MHz, DMSO-*d*_6_, ppm, δ): 176.72, 172.84, 67.07, 57.13, 56.36, 41.38.

##### Tris(2-hydroxyethyl)ammonium Dihydrogen Citrate [TEA][DHCIT]

Reagents: tris(2-hydroxyethyl)ammine (16.15 g, 107.17 mmol) and citric acid (20.80 g, 107.17 mmol). Reaction time: 1 h. The reaction product was obtained as a highly viscous syrup-like substance. Yield ≥99%. ^1^H NMR (400 MHz, DMSO-*d*_6_, ppm, δ): 3.68 [t, 6H, *J* = 5.5 Hz, C*H*_2_OH], 3.13 [t, 6H, *J* = 5.5 Hz, NC*H*_2_], 2.59 [d, 1H, *J* = 15.2 Hz, C*H*_α_C = O], 2.52 [d, 1H, *J* = 15.2 Hz, C*H*_β_C = O]. ^13^C NMR (100.6 MHz, DMSO-*d*_6_, ppm, δ): 177.27, 172.03, 72.06, 56.50, 56.03, 44.61.

##### Tris(2-hydroxyethyl)ammonium Chloride [TEA]Cl

Reagents: tris(2-hydroxyethyl)ammine (1.82 g, 12.07 mmol) and hydrochloric acid (37% solution in water) (1.01 mL, 12.07 mmol). Reaction time: 5 min. Yield ≥99%. ^1^H NMR (400 MHz, DMSO-*d*_6_, ppm, δ): 3.73 [t, 6H, C*H*_2_OH], 3.23 [t, 6H, *J* = 7.3 Hz, NC*H*_2_]. ^13^C NMR (100.6 MHz, DMSO-*d*_6_, ppm, δ): 57.03, 56.03

### 2.2 Apparatus and Procedure

The glass material employed in the synthetic reactions was dried in an oven at 333 K for 24 h before use. The evolution of the reactions was monitored by t.l.c. employing silica-gel sheets (Merck, TLC Silica gel 60 F254).

Spectroscopic data were provided by the Center of Scientific-Technological Support to Research (CACTI) of the University of Vigo. ^1^H and ^13^C NMR spectra were recorded on a BRUKER ARX 4CO spectrometer at 400.1621 (^1^H) and 100.6314 (^13^C) MHz, respectively. DMSO-d6 for NMR (Thermo Scientific, 99.9 atom % D) was employed as deuterated solvent. Chemical shifts are quoted in parts per million (ppm) relative to the signals corresponding to the residual nondeuterated solvents (DMSO: δH = 2.51 ppm, δC = 39.89 ppm). Lyophilization was carried out in a Telstar LyoQuest apparatus; samples were left for 24 h at −86.3 °C and 0.042 mbar.

Thermal events for PILs were determined with a TA Instruments Q2000 differential scanning calorimeter (DSC) equipped with an RCS 90 refrigerated cooling system. Calibration was carried out with indium for temperature and with sapphire references for the cell constant. Hermetic aluminum pans and lids were used for calibration and measurements. An empty pan was used as reference, and dry nitrogen (Nippon Gases, 99.999%) at a flow rate of 50 mL/min was used as purge gas. The thermal program consisted of an initial heating up to 353 at 5 K/min. Afterward three cycles were performed: cooling down to 183 at 5 K/min, 10 min isotherm, heating up to 353 at 5 K/min and 10 min isotherm. Melting temperatures were determined at the onset of the endothermic peaks and glass transitions as the midpoint of the sigmoidal portion of the thermogram resulting from the variation in heat capacity, with the software Universal Analysis 2000, version 4.5.0.5 by TA Instruments.

Thermal stability of the PILs was determined in a TA Instruments Q500 thermogravimetric analyzer (TGA). Nitrogen (Praxair, 99.999%) was used as balance (flow rate of 40 mL/min) and sample (flow rate of 60 mL/min) purge gas. The thermal program consisted of heating the sample up to 773 at 5 K/min. The software Universal Analysis 2000, version 4.5.0.5 by TA Instruments, was used. In order to obtain a more rigorous information about the range of temperatures in which the use of these PILs is safe (without decomposition or vaporization), isothermal TGA studies were also performed (with a duration of 12 h) at different temperatures.

### 3.1 Results and Discussion

#### 3.1.1 Synthesis

Keeping tris(2-hydroxyethyl)ammonium [TEA] as the cation, the selected anions were lactate [LAC], hydrogen succinate [HSUC], hydrogen malate [HMAL], and dihydrogen citrate [DHCIT]. [TEA]Cl was also synthesized for ionization studies. The structures are shown in Figure 1.

The synthesis of the selected TEA-based PILs was initially performed by applying the usual methodology described in literature.^3,11−14^ That is, an equimolecular amount of tris(2-hydroxyethyl)amine was treated with the corresponding acid in a solvent (MeOH in this case) at room temperature for 24 h with constant stirring. The reaction product was then heated in vacuum to eliminate the solvent and water. However, our results showed the formation of some impurities when the reaction product was heated, in all cases. This problem was previously observed with similar PILs,^15^ however, it is frequently neglected in the preparation of these compounds. The impurities are likely due to the formation of condensation products associated with the dehydration of the salt to corresponding amide.^16^ Decomposition increased with increased temperature and duration of heating. To avoid this drawback, optimization of the chemical reactions was conducted by applying different conditions:

1. First, the reactions were carried out employing the smallest amount of MeOH needed to dissolve the reagents, heating with stirring at 323 K until the end of the reaction. Monitoring of the reaction progress by t.l.c. showed that after 1 h the reactions were completed in all cases. The NMR spectra of the crude reaction mixtures showed MeOH as the only impurity. However, when heat was applied to eliminate the solvent, condensation impurities began to appear.
2. The reactions were then tried in absence of solvent, by heating to the melting point of the acids when they were a solid (succinic, malic and citric acid). This allowed the completion of the reactions in short times (from 15 to 40 min depending on the acid), but the presence of condensation impurities were also observed by NMR.
3. Finally, the reactions were performed without solvent, as previously proposed,^17−19^ by heating at 323 K. Monitoring of the reaction by t.l.c. showed that after short times (from 15 to 60 min depending on the acid) the reactions were completed to give very pure salts (see NMR spectra in SI Figures S1–S8). To avoid condensation reactions by heating, the obtained salts were freeze-dried by lyophilization. Table 1 shows names, abbreviations, purities, and water content of the synthesized salts.

**Table 1:** Synthesized Salts

name	abbreviated name	CAS	mass fraction purity (%)^a^	analysis	water content (wt %)^b^
tris(2-hydroxyethyl)ammonium lactate	[TEA][LAC]	20475-12-1	≥99	NMR, t.l.c.	0.2
tris(2-hydroxyethyl)ammonium hydrogen succinate	[TEA][HSUC]		≥99	NMR, t.l.c.	0.2
tris(2-hydroxyethyl)ammonium hydrogen malate	[TEA][HMAL]		≥99	NMR, t.l.c.	0.7
tris(2-hydroxyethyl)ammonium dihydrogen citrate	[TEA][DHCIT]		≥99	NMR, t.l.c.	0.7
tris(2-hydroxyethyl)ammonium chloride	[TEA]Cl	637-39-8	≥99	NMR, t.l.c.	0.02

a See degree of ionization in Table 2

b Determined by Karl Fischer titration in a Metrohm 899 coulometer.

#### 3.1.2 Ionicity

The properties exhibited by PILs mainly depend on their ionicity, as well as on the possible hydrogen bonds that can be formed. Even though this parameter is frequently neglected in literature regarding this type of ILs, it is essential to know their ionicity to explain their behavior and properties.

In general, it is estimated that a protic ionic liquid is a salt when the proton transfer is at least 99%.^2^ Some authors have used p*K*_a_ values to estimate the degree of proton transfer in PILs. It has been suggested that the relative difference in aqueous p*K*_a_ can provide some measure of proton transfer. A PIL with a large Δp*K*_a_^aq^ would predicted to be more “ionic” than a PIL with a small one. However, it has been shown that Δp*K*_a_^aq^ > 10 seems to be required for the full proton transfer to occur in a PIL.^20,21^ This means that a high value of Δp*K*_a_^aq^ is required to provide an adequate prediction. These results suggest that the IL solvation environment can have a very strong effect on the degree of proton transfer, and that the Δp*K*_a_^aq^ would be a relatively poor estimation of proton transfer degree in the case of the PILs under study. For this reason, other methodologies are needed.

In this context, NMR spectroscopy has shown to be particularly useful^22^ to determine the percentage of ionicity from the chemical shifts of the free base (B), the base hydrochloride (BH^+^) and the PIL, through the following equation:^23^
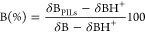
1Quantum-chemical calculations have shown that triethanolamine can form both ionic complexes (BH^+^A^–^) and H-bonding molecular complexes (B···HA) when is treated with an acid.^18,24^ This has been confirmed in some literature data on the properties of salts based on triethanolamine with carboxylic, sulfonic and inorganic acids.^19^

The NMR methodology described above was applied in order to determine the ionicity of the TEA PILs synthesized in this work. The results are shown in Table 2.

**Table 2:** Chemical Shifts (ppm) of NCH_2_ Protons for B, B.HCl, and PILs in DMSO-d_6_ as well as Ionicity (%) and Δp*K*_a_^aq^ Values Determined at 298.15K and 0.1 MPa

compound	δNCH_2_	ΔδNCH_2_	ionicity (%)	Δp*K*_a_^aq^
triethanolamine	3.41		0	
[TEA]Cl	3.23	0.69	100	14
[TEA][LAC]	2.70	0.16	23	3.9
[TEA][HSUC]	2.68	0.14	20	3.6
[TEA][HMAL]	3.02	0.48	70	4.4
[TEA][DHCIT]	3.13	0.59	86	4.6

As shown in Table 2, the percentage of ionised species is high in [TEA][DHCIT] and [TEA][HMAL] (86 and 70% respectively), but low in [TEA][LAC] and [TEA][HSUC] (23% and 20%, respectively). So, it can be said that while [TEA][DHCIT] and [TEA][HMAL] are mainly composed of ions, [TEA][LAC] and [TEA][HSUC] are mostly H-bonded molecular complexes. It is interesting to note that in this series the percentage of ionicity moves in the same way that Δp*K*_a_^aq^ (see Table 2): as the Δp*K*_a_^aq^ value increases the ionicity (%) increases.

#### 3.1.3 Phase Transitions

**Figure 2:**
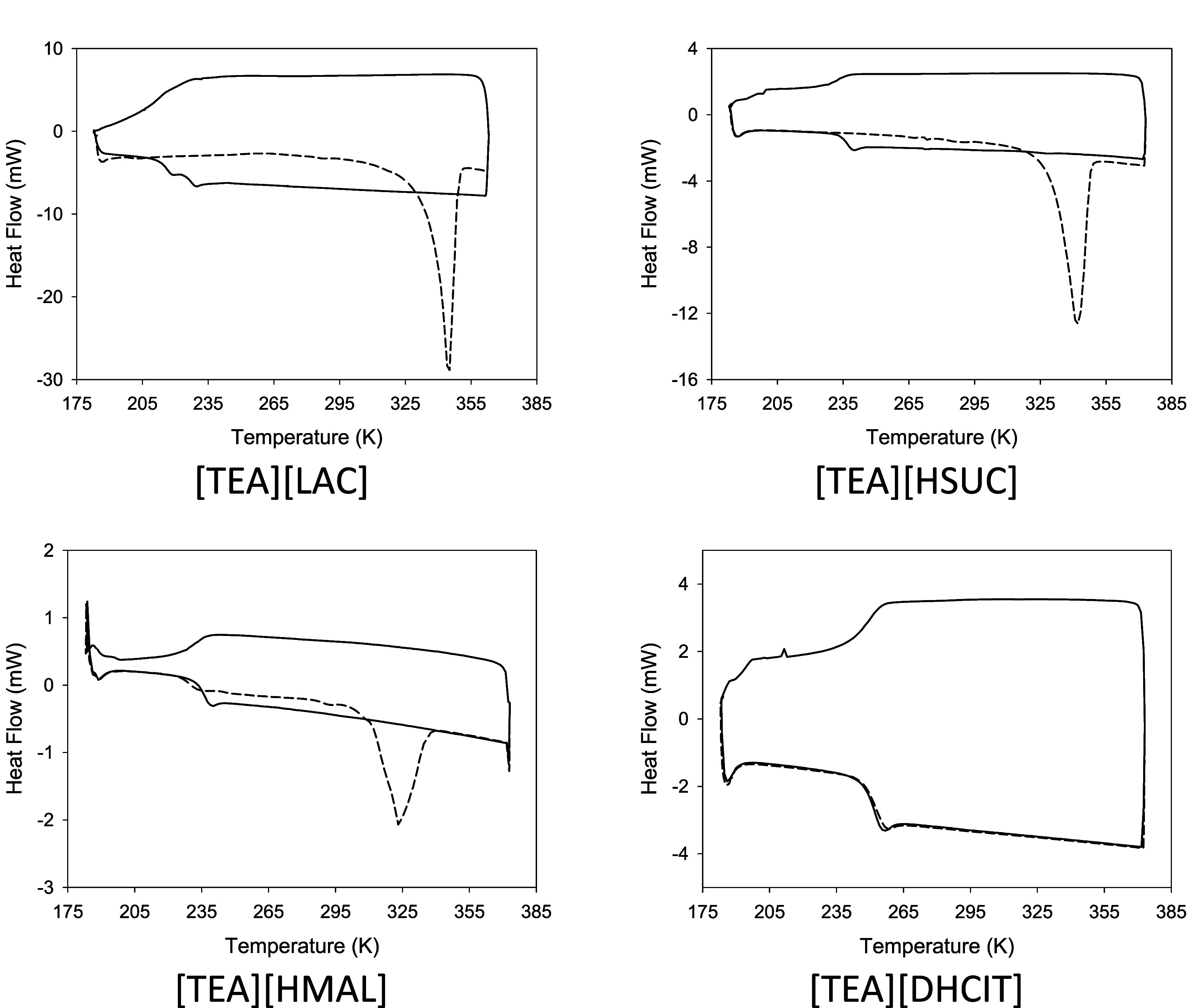
DSC thermograms of synthesized compounds: First run (- - - -), Third run (—).

First and third DSC cycles of [TEA][LAC] are presented in Figure 2. In the first heating cycle, a melting transition appears at 336 K (see Table 3). However, at the cooling rate used (5 K/min), crystallization was not observed. In the subsequent cycles, a glass transition temperature is observed in the cooling cycle at 209 K. Nonetheless, in the heating cycle, the compound undergoes glass transition in two steps, covering a range of temperature from 216 to 227 K. As the degree of ionization found for this PIL was very low (23%), the reason of this phenomenon could be associated to the glass transition of triethanolamine, determined to be 207 K. Moreover, when mixed triethanolamine with lactic acid, the glass transition of the former appeared at higher temperatures. So, the two-steps glass transition of this [TEA][LAC] could be related to the presence of the neutral species. However, this phenomenon was not found in the case of the other PILs. There is not much agreement in the literature^3,11,13^ regarding the final phase (solid or liquid) or the thermal events of [TEA][LAC]. Yuan et al.^3^ carried out the synthesis by neutralization of ethanolamine in ethanol with the acid. A viscous liquid was obtained and a glass transition at 213.8 K (heating rate 10 K/min) was identified in the DSC. This value is in relatively good agreement with our result because it is well-known that the cooling/heating rates affect glass transition temperatures. Faraji et al.^13^ detected the glass transition at about 223 K and a peak at 273 K related to the melting point of water. The high water content of the lactate in these two works could be the reason for their liquid state. However, Pavlovica et al.,^11^ using water as solvent in the synthesis of the [TEA][LAC], obtained a solid as in our case and identified a melting point at 318–319 K but the method to determine it is unclear.

**Table 3:** Thermal Characterisation of PILs at 0.1 MPa^a^

compound	*T*_m,onset_ (K)	*T*_g_ (K)	*T*_0_ (K)	*T*_d,onset_ (K)	*T*_d,5% onset_ (K)	*T*_max_ (K)
[TEA][LAC]	336^b^	216–227/209^d^,^c^	372	460	423	516, 627, 653
[TEA][HSUC]	331^b^	238^c^	373	429	429	460, 603, 628
[TEA][HMAL]	309^b^	234^b^, 236^c^	373	459	423	492, 551, 611
[TEA][DHCIT]		251^b^,^c^	373	443	426	468, 548, 613

a *u*(*T*) = 1 K, *u*(*P*) = 5 kPa.

b First cycle.

c Subsequent cycles.

d Cooling ramp.

In the case of [TEA][HSUC], we obtained a white solid and a melting transition was observed at 331 K in the first heating ramp (see Table 3). In the subsequent cycles, only a glass transition temperature was detected (238 K). Fundamensky et al.,^14^ using methanol as solvent in the synthesis, carried out a DSC (at temperatures higher than 273.15K) and found a melting point at 346.86 K. Heating and cooling rates, along with the presence of impurities, mainly water, drastically affect thermal events of these PILs. [TEA][HMAL] and [TEA][DHCIT] were synthesized in this work for the first time. In the case of the hydrogen malate PIL, in the first cycle, glass and melting transitions were detected at 234 and 309 K, respectively. In the third cycle, only a glass transition was observed at 236 K. In the case of [TEA][DHCIT], a highly viscous syrup-like substance, a glass transition was found at 251 K in all DSC cycles.

#### 3.1.4 Thermogravimetric Analysis

**Figure 3:**
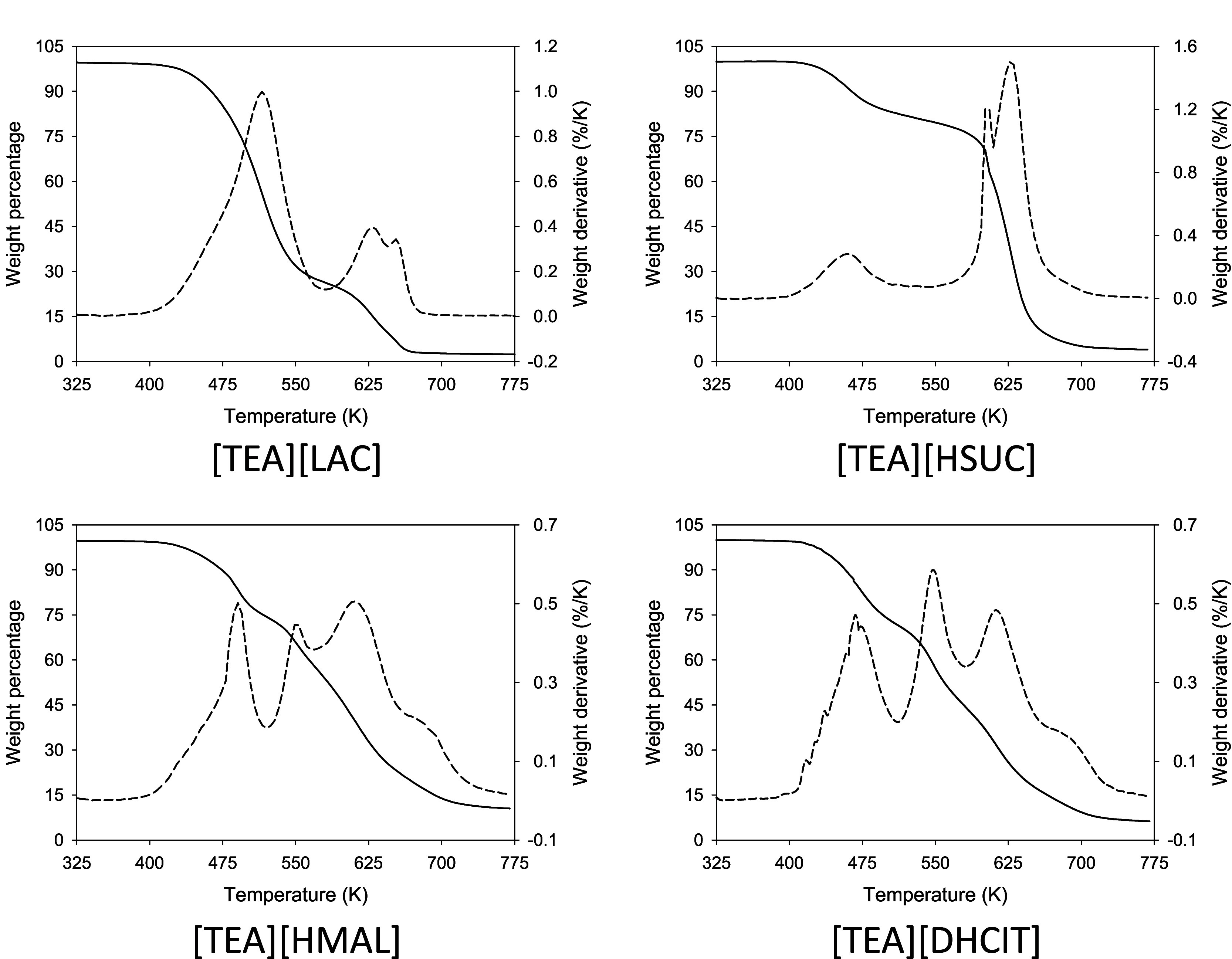
TGA thermograms of synthesized compounds.

Short-term thermal stability of synthesized PILs was determined by dynamic TGA studies. Results are shown in Figure 3. Characteristic two step decomposition processes were observed for all the compounds. The derivative of the thermograms reveals three peaks for all the PILs, the first one associated with the first step and the other two linked with the second step, that are related with the maximum decomposition rate of neutral and ionic species. Moreover, as salts under study show percentages of ionicity between 20% and 86%, the presence of acid and base species is significant and the contribution to mass loss is due not only to decomposition but also to vaporization of the neutral forms. Table 3 shows the temperature at which the PILs start to decompose (*T*_0_), the onset temperature at the first decomposition step (*T*_d,onset_), the onset temperature at the 5% weight lost (*T*_d,5% onset_), and temperatures of maximum mass loss rate (*T*_max_). The ionicity of the compound does not seem to significatively affect *T*_0_ or *T*_d,5% onset_. Moreover, these parameters are little affected by the anion.

**Figure 4:**
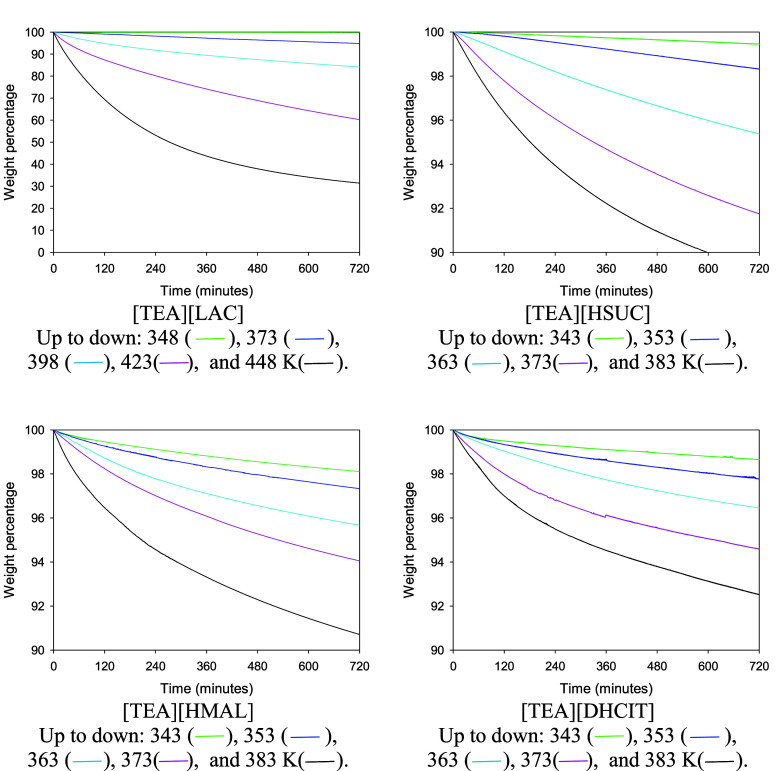
Isothermal TGA curves of synthesized compounds.

Isothermal TGA runs with a duration of 12 h were also performed at different temperatures in order to determine long-term thermal stability of the synthesized compounds. Results are shown in Figure 4. It can be observed that all the PILs lose weight at temperatures notably lower than *T*_d,5% onset_, even at temperatures lower than 350 K. This gives values less optimistic of the thermal stability obtained by dynamic measurements by ourselves or reported for [TEA][LAC] by Yuan et al.^3^ (*T*_d_ = 511.2 K) and for [TEA][HSUC] by Fundamensky et al.^14^ (*T*_d5%_*=* 461.15K).

## 4 Conclusions

In this work, tris(2-hydroxyethyl)ammonium PILs were synthesized with lactate, hydrogen succinate, hydrogen malate, and dihydrogen citrate anions. It was found that the usually applied methods of synthesis for this type of PILs, involving the use of solvents or heating at the melting point of the acids, lead to the appearance of impurities. To avoid condensation reactions associated with heating, working at moderate temperatures and in absence of solvents is proposed.

Percentages of ionicity between 20% ([TEA][HSUC]) and 86% ([TEA][DHCIT]) were found. Thus, the widely used term “ionic liquid” for these systems that are mixtures of neutral and ionic species is not rigorous and must be used with caution. Moreover, PILs should not be considered just one compound and should always be characterized through their ionicity.

[TEA][LAC], [TEA][HSUC], and [TEA][HMAL] showed a melting point in the first DSC cycle, and all the PILs showed a glass transition after first heating–cooling cycle. Relatively low thermal stabilities, with *T*_d,5% onset_ between 423 and 426 K, were found. The influence of the anion on the thermal stability was found to be low. Isothermal TGA showed that mass loss associated with these compounds starts at temperatures close to 350 K.

## Supporting Information Available

[TEA][LAC] ^1^H NMR spectrum, [TEA][LAC] ^13^C NMR spectrum, [TEA][HSUC] ^1^H NMR spectrum, [TEA][HSUC] ^13^C NMR spectrum, [TEA][HMAL] ^1^H NMR spectrum, [TEA][HMAL] ^13^C NMR spectrum, [TEA][DHCIT] ^1^H NMR spectrum, and [TEA][DHCIT] ^13^C NMR spectrum (PDF)
