# Supplementary material for: Tris(2-hydroxyethyl)ammonium-Based Protic “Ionic Liquids”: Synthesis and Characterization
Source: J Chem Eng Data. 2024 Apr 24;69(10):3427–34. doi: 10.1021/acs.jced.4c00024 (PMC11480899; doi:10.1021/acs.jced.4c00024)
Supplement: Supplementary file 1 — je4c00024_si_001.pdf [file je4c00024_si_001.pdf]

J. Chem. Eng. Data

Tris(2-hydroxyethyl)ammonium based protic “ionic liquids”: Synthesis and characterisation

Emilia Tojo[1], Alexandra Cáceres[2], Alba Somoza[2], Carlos A. Pena[2], Ana Soto[2*]

[1] Department of Organic Chemistry, Faculty of Chemistry, Universidade de Vigo, 36210 Vigo, Spain

[2] CRETUS. Department of Chemical Engineering, Universidade de Santiago de Compostela, E-15782, Santiago de Compostela, Spain.

Supplementary Information

* Corresponding author

E-mail address: ana.soto@usc.es

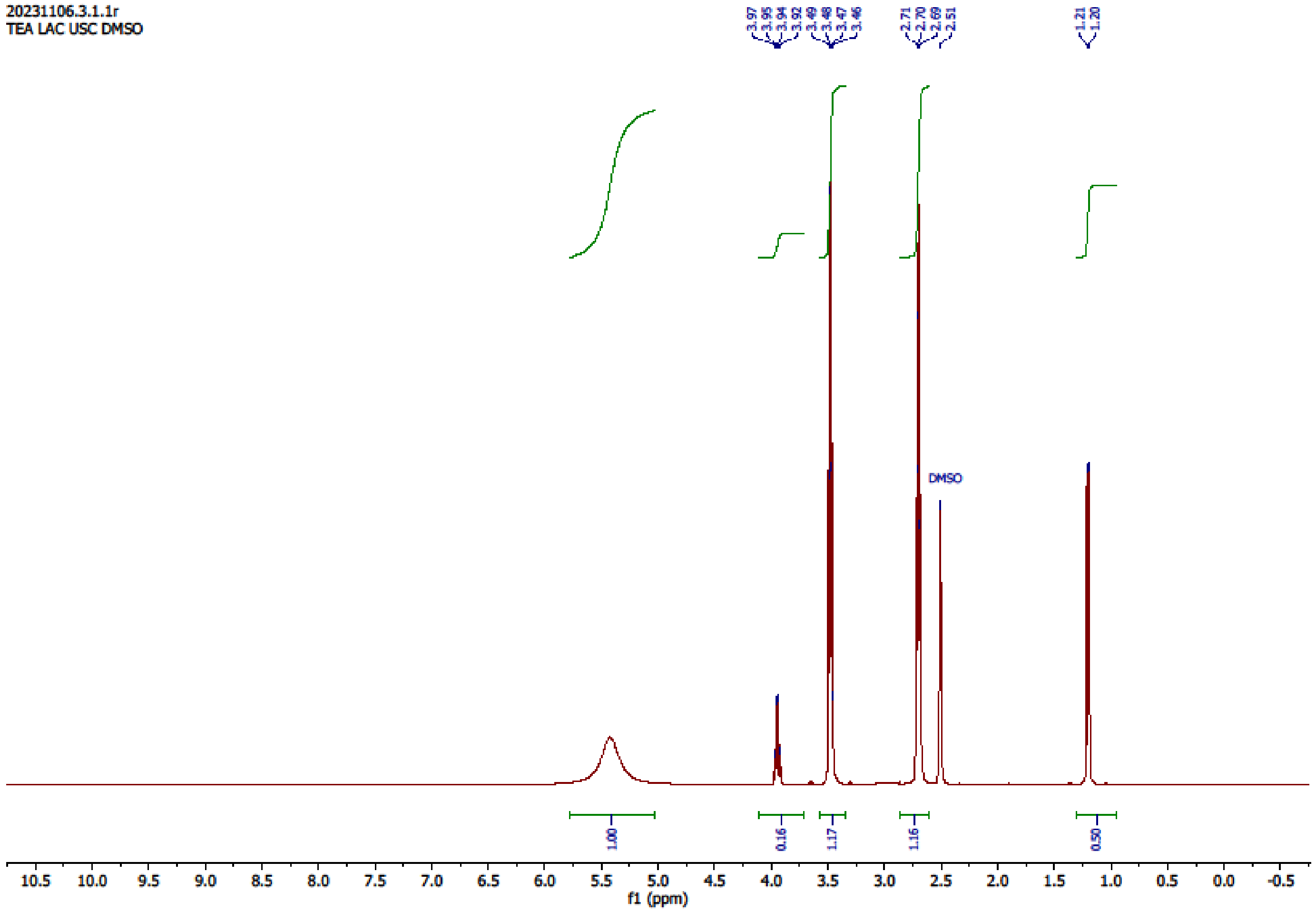

Fig. S1. [TEA][LAC] ^{1}H NMR spectrum

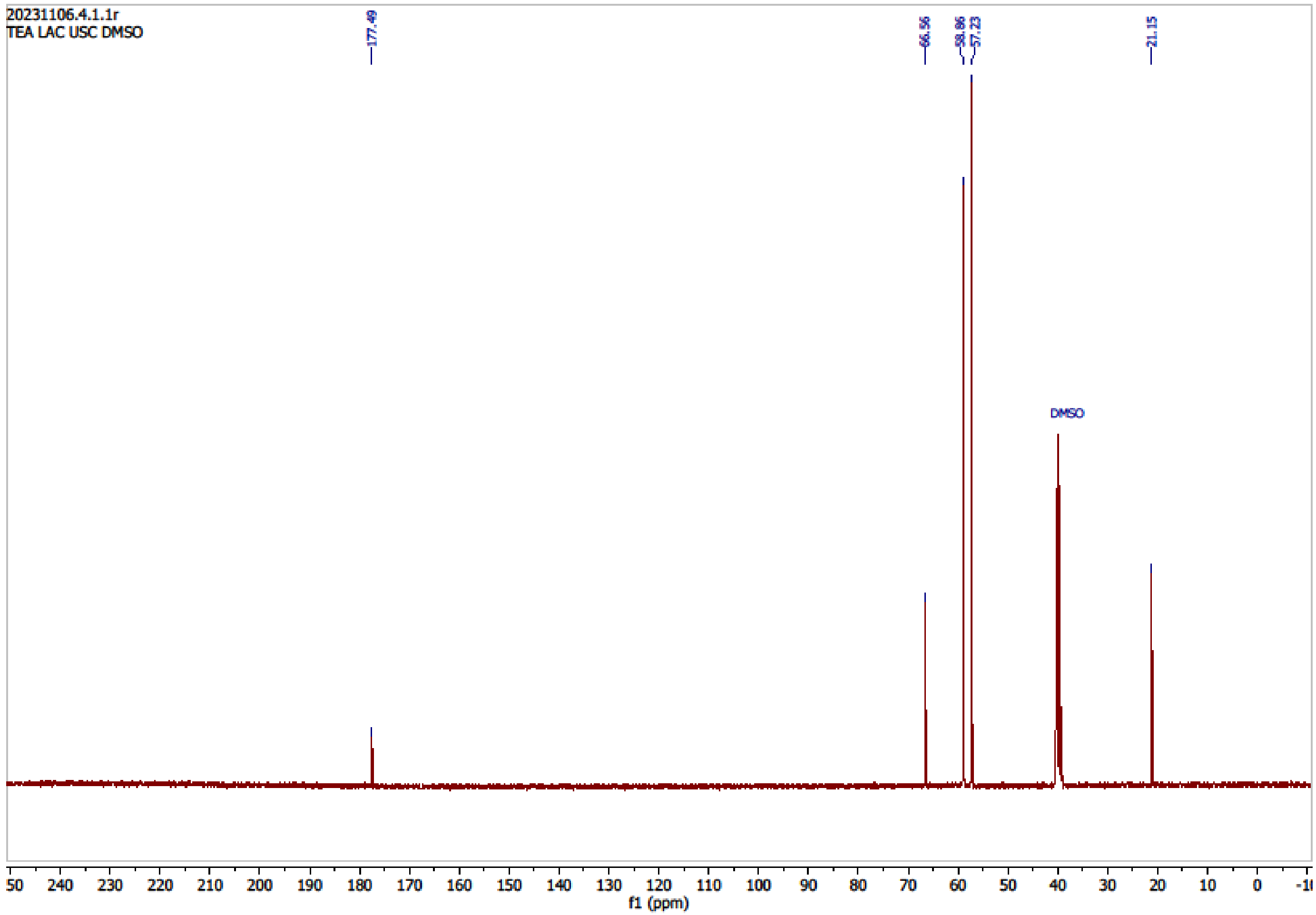

Fig. S2. [TEA][LAC] ^{13}C NMR spectrum

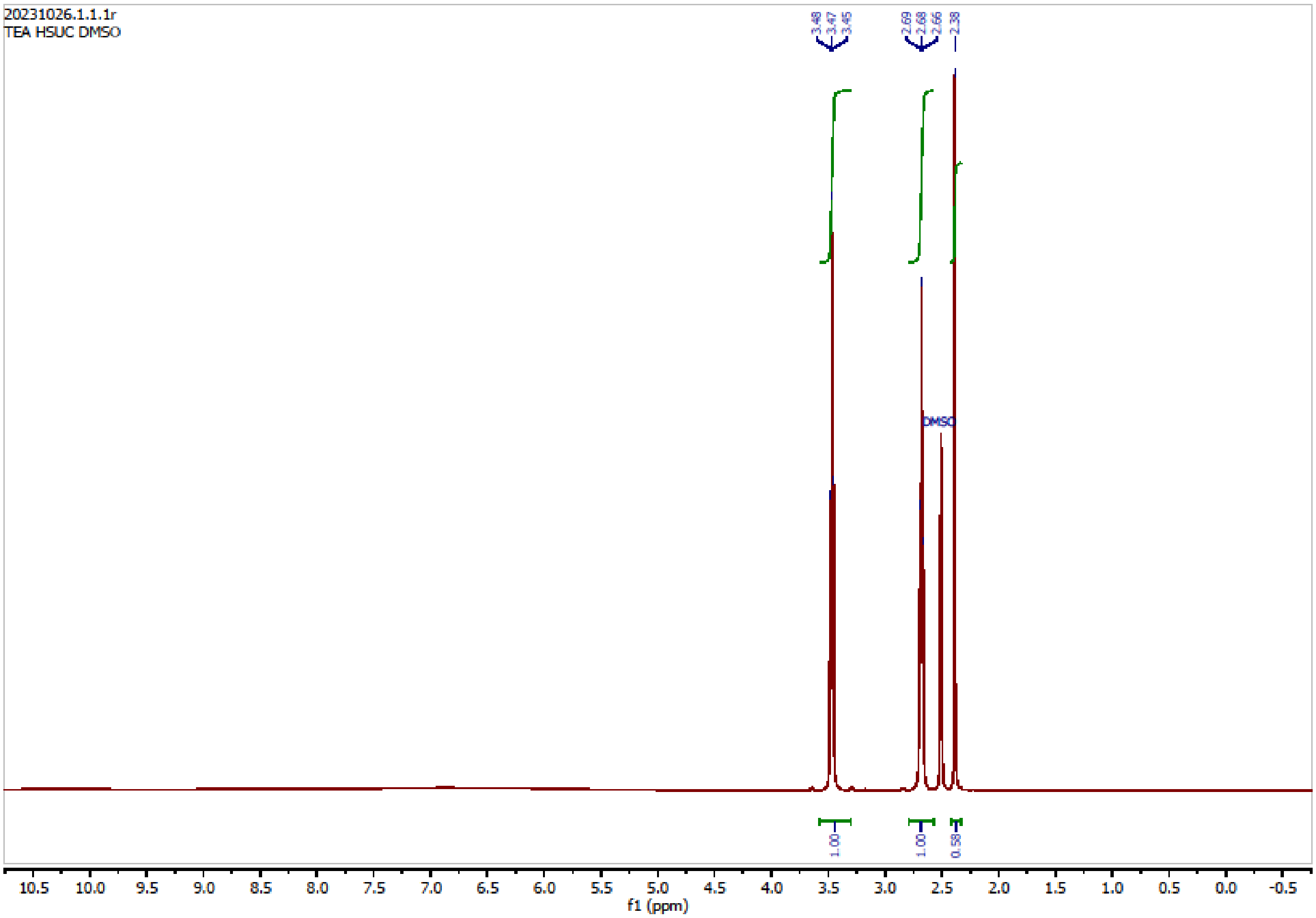

Fig. S3. [TEA][HSUC] ^{1}H NMR spectrum

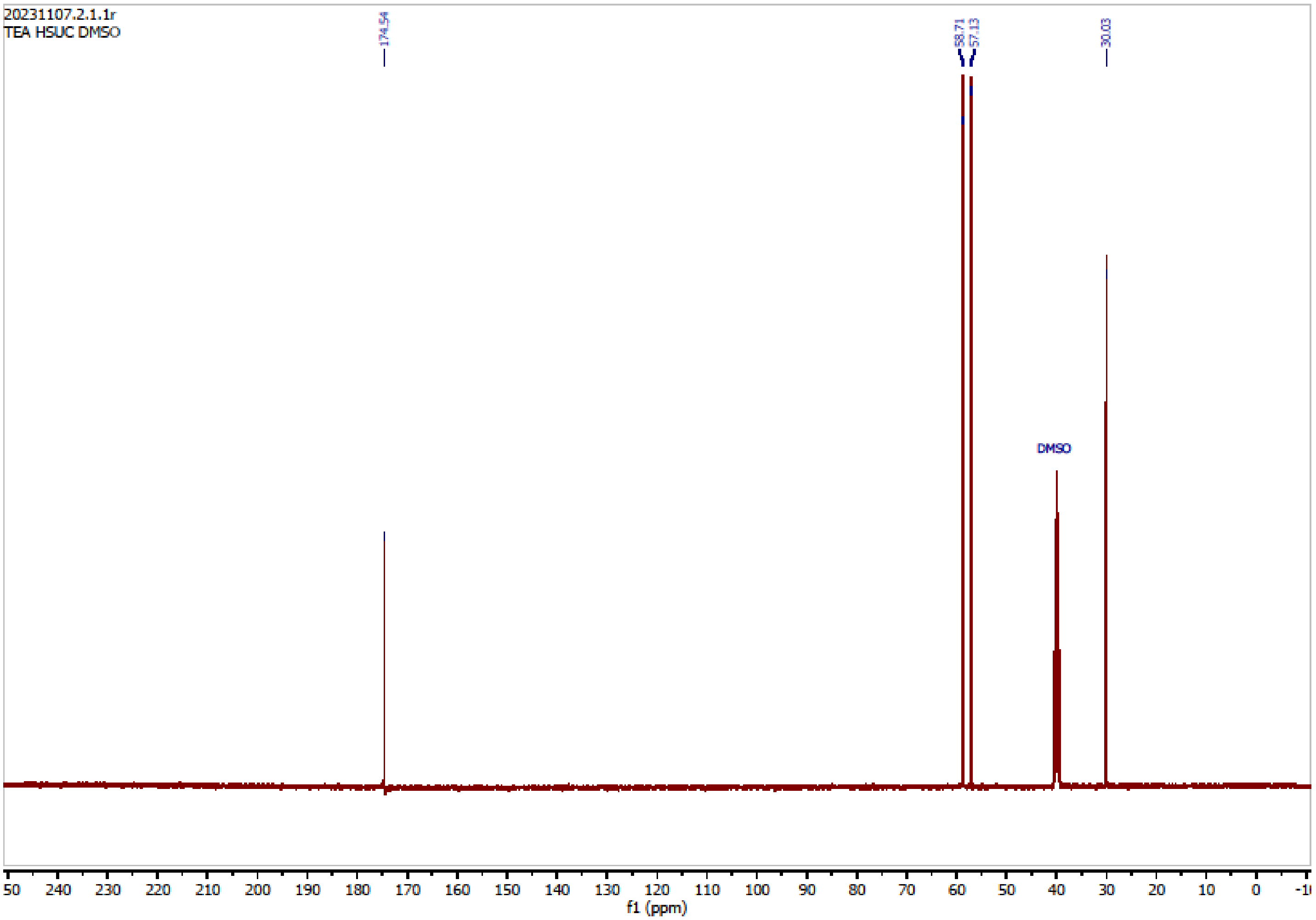

Fig. S4. [TEA][HSUC] ^{13}C NMR spectrum

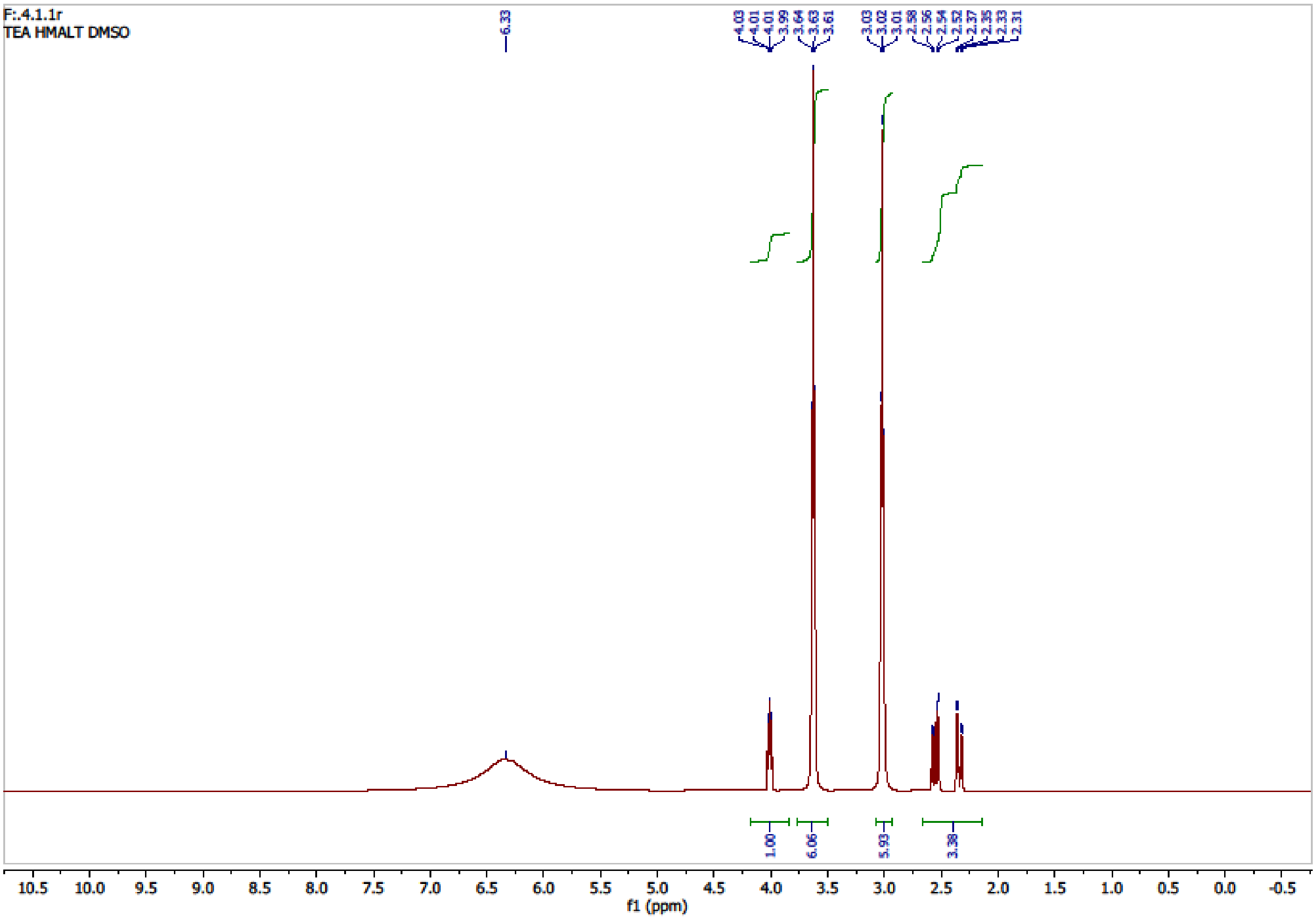

Fig. S5. [TEA][HMAL] ^{1}H NMR spectrum

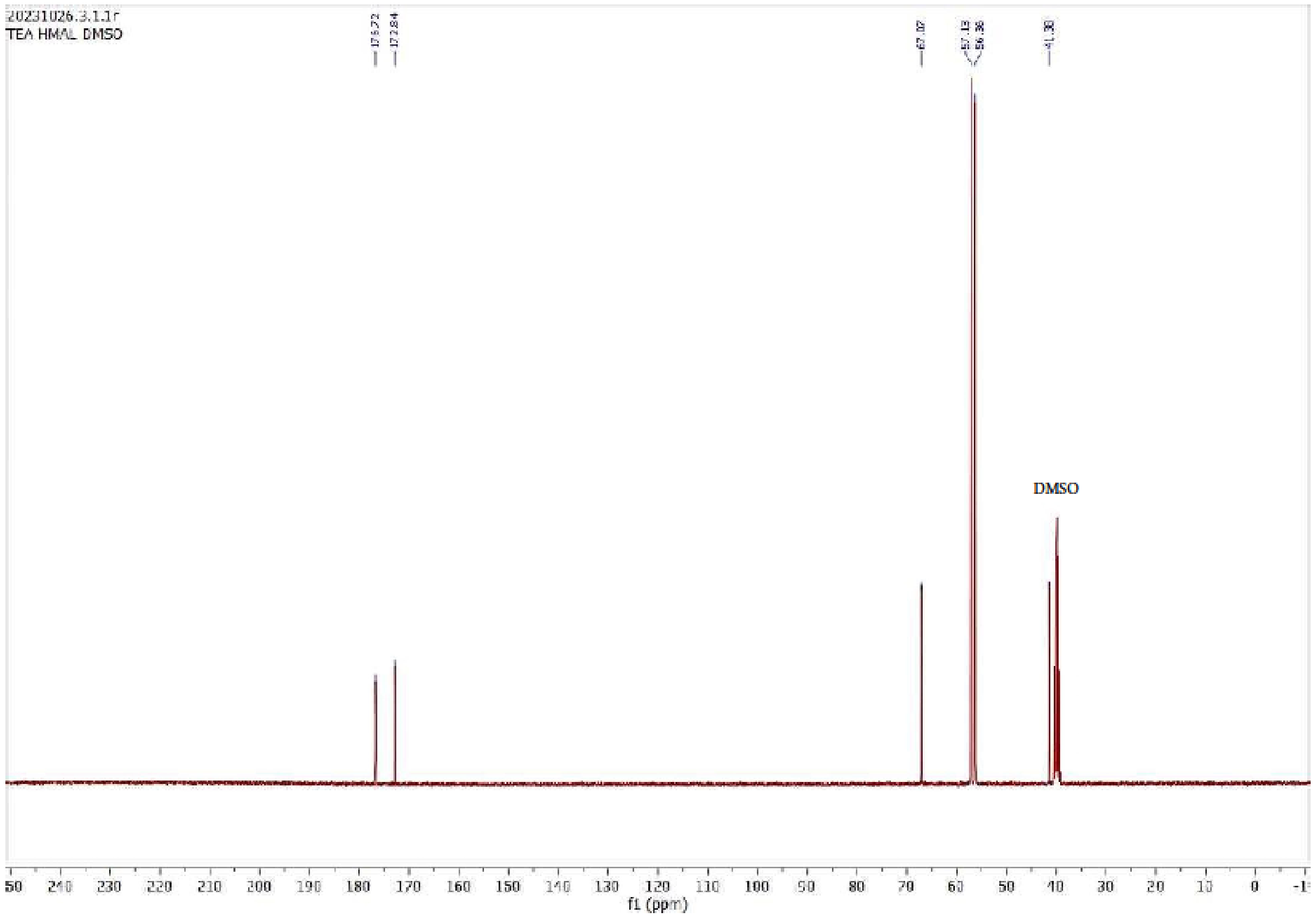

Fig. S6. [TEA][HMAL] ^{13}C NMR spectrum

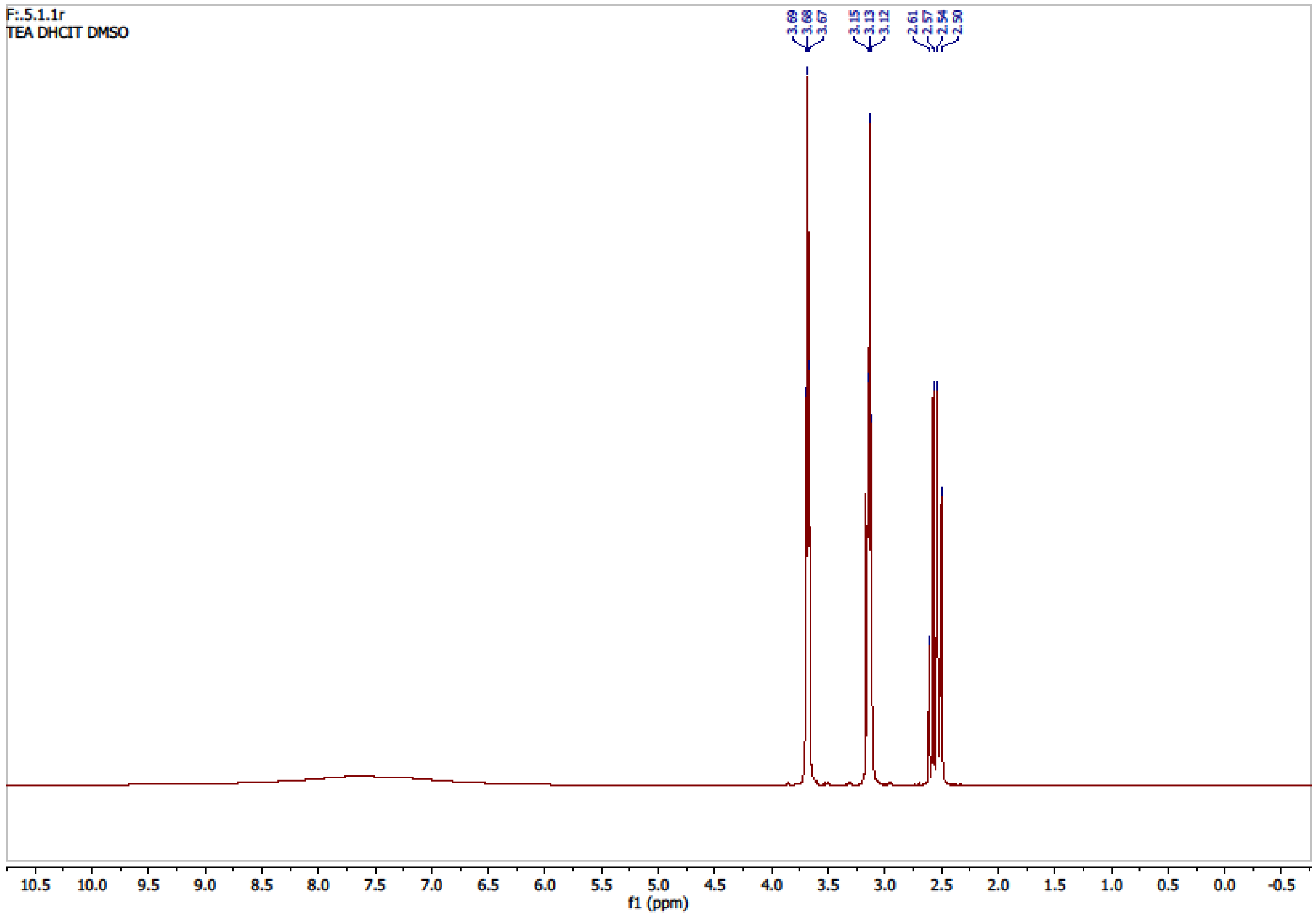

Fig. S7. [TEA][DHCIT] ¹H NMR spectrum

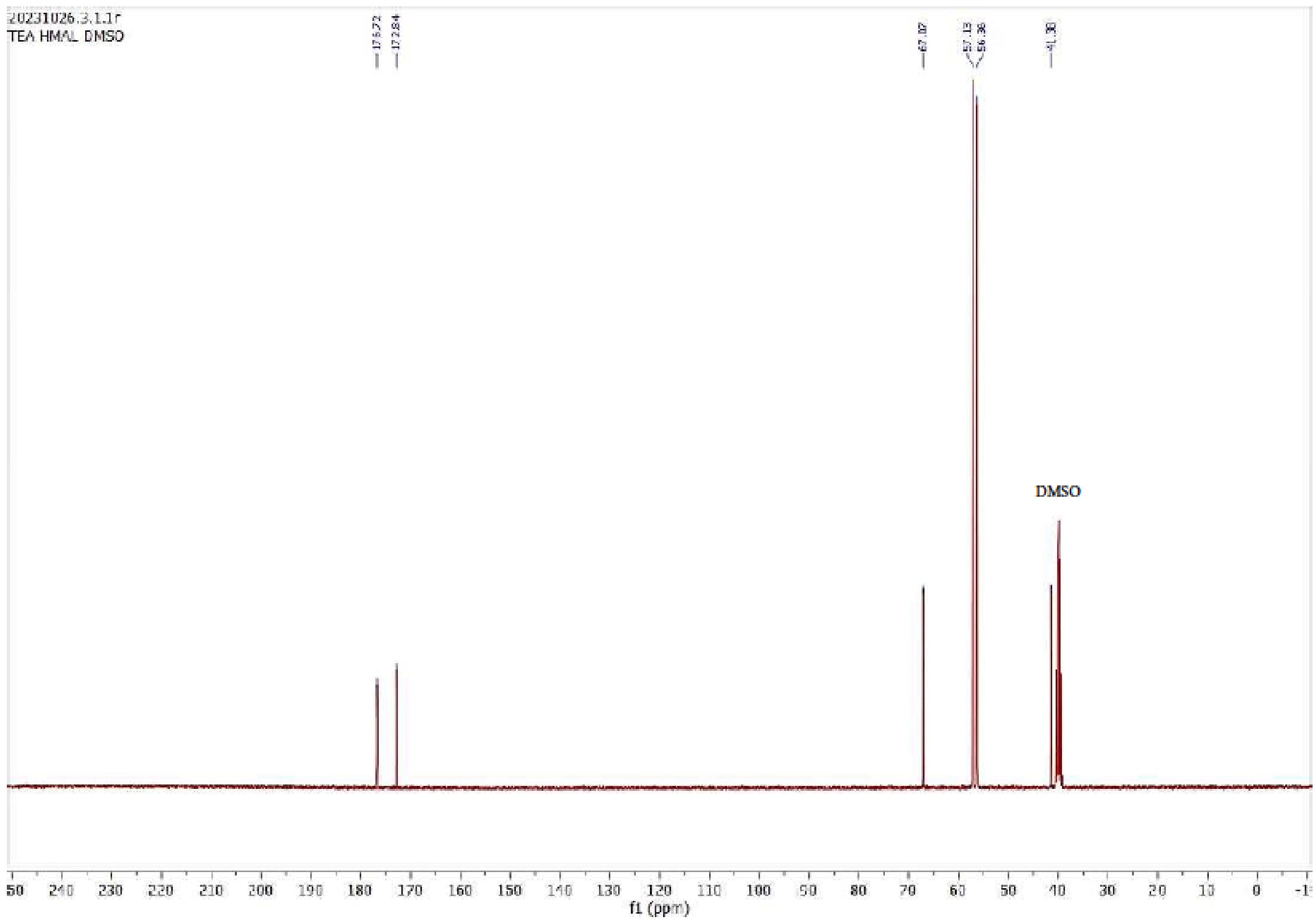

Fig. S8. [TEA][DHCIT] ^{13}C NMR spectrum
